# Real-time analysis and visualization of nanopore metagenomic samples with MARTi

**DOI:** 10.1101/gr.280550.125

**Published:** 2025-11

**Authors:** Ned Peel, Samuel Martin, Darren Heavens, Douglas W. Yu, Matthew D. Clark, Richard M. Leggett

**Affiliations:** 1 Earlham Institute, Norwich, NR4 7UZ, United Kingdom;; 2 University of East Anglia, Norwich, NR4 7TJ, United Kingdom;; 3 Yunnan Key Laboratory of Biodiversity and Ecological Security of Gaoligong Mountain and Kunming Institute of Zoology, Chinese Academy of Sciences, Kunming, China 650201;; 4 Centre for Microbial Interactions, Norwich, NR4 7UG, United Kingdom;; 5 Natural History Museum, London, SW7 5BD, United Kingdom

## Abstract

The emergence of nanopore sequencing technology has the potential to transform metagenomics by offering low-cost, portable, and long-read sequencing capabilities. Furthermore, these platforms enable real-time data generation, which could significantly reduce the time from sample collection to result, a crucial factor for point-of-care diagnostics and biosurveillance. However, the full potential of real-time metagenomics remains largely unfulfilled due to a lack of accessible, open-source bioinformatic tools. We present Metagenomic Analysis in Real-Time (MARTi), an innovative open-source software designed for the real-time analysis, visualization, and exploration of metagenomic data. MARTi supports various classification methods, including BLAST, Centrifuge, and Kraken2, letting users customize parameters and utilize their own databases for taxonomic classification and antimicrobial resistance analysis. With a user-friendly, browser-based graphical interface, MARTi provides dynamic, real-time updates on community composition and AMR gene identification. MARTi's architecture and operational flexibility make it suitable for diverse research applications, ranging from in-field analysis to large-scale metagenomic studies. Using both simulated and real-world data, we demonstrate MARTi's performance in read classification, taxon detection, and relative abundance estimation. By bridging the gap between sequencing and actionable insights, MARTi marks a significant advance in the accessibility and functionality of real-time metagenomic analysis.

Metagenomics is revolutionizing our understanding of the diversity and ecology of ecosystems in environmental and even in clinical settings. Advances in this field are largely driven by developments in DNA sequencing technology and the associated analysis tools and pipelines (Escobar-Zepeda et al. 2015). In contrast to the relatively bulky and expensive sequencing platforms developed by Illumina and Pacific Biosciences (PacBio), the MinION, by Oxford Nanopore Technologies (ONT), is a low-cost, portable sequencing device that almost any research group has the resources to acquire and operate. The technology is becoming increasingly used for detecting and characterizing pathogenic organisms (Taxt et al. 2020), outbreak surveillance (Quick et al. 2016; Yakovleva et al. 2022), and in situ sequencing (Burton et al. 2020; Latorre-Pérez et al. 2021), that is, taking the sequencer to the sample. Significantly, the ONT platform is the first to enable true real-time analysis, with reads able to be accessed during a sequencing run (Leggett and Clark 2017), which, combined with the manufacturers’ API, enables focusing of sequencing on specific species within a metagenomic sample using “adaptive sampling” (Martin et al. 2022). Real-time analysis could facilitate a much faster route from sample to results in time-critical situations such as point-of-care diagnostics and biosurveillance. However, the full potential of real-time sequencing remains largely unrealized due to the lack of open-source, offline analysis tools.

ONT's own EPI2ME platform is currently the most well-known example of a real-time metagenomic analysis tool. Initially, ONT developed the EPI2ME Agent software, which featured several cloud-based analysis workflows including two popular metagenomic pipelines: What's In My Pot (WIMP) and Antimicrobial Resistance Mapping Application (ARMA) (Juul et al. 2015). WIMP classified reads using Centrifuge (Kim et al. 2016) with a database of bacterial, viral, and fungal RefSeq genomes and presented a taxonomic tree view of the sample. ARMA used minimap2 (Li 2018) to align reads against all reference sequences available in the Comprehensive Antibiotic Resistance Database (CARD) to identify antimicrobial resistance (AMR) genes (Alcock et al. 2020). However, due to EPI2ME Agent's closed nature, lack of flexibility or customization, including classification parameters and choice of reference databases, and the need for a fast and stable internet connection, EPI2ME Agent was unsuitable for many in-field sequencing applications. Subsequently, ONT released the EPI2ME Labs tool (now simply called “EPI2ME”), a collection of Nextflow workflows that can be run either locally or in the cloud. This includes wf-metagenomics, a taxonomic classification pipeline that can use either Kraken2 (Wood et al. 2019) or minimap2 with a user-provided database. Despite these advancements, EPI2ME still has limited data visualization and data export capabilities and no between-run comparison features.

Here, we present Metagenomic Analysis in Real-Time (MARTi), an open-source software tool that enables real-time analysis, visualization, and exploration of metagenomic sequencing data. MARTi allows users to choose a classification method (Kraken2, Centrifuge, BLAST [Camacho et al. 2009], or DIAMOND [Buchfink et al. 2021]), customize classification parameters, and provide their own databases. As an offline tool with low memory options, MARTi can be used for in-field taxonomic composition and AMR gene analysis in real-time on a standard laptop. MARTi can also carry out larger scale, complex analyses using a high performance computing (HPC) cluster. Finally, MARTi features an intuitive browser-based GUI that facilitates exploration of metagenomic results and enables intuitive side-by-side sample comparison on a laptop/desktop, tablet, or smartphone.

## Results

### Software architecture

**Figure 1.**
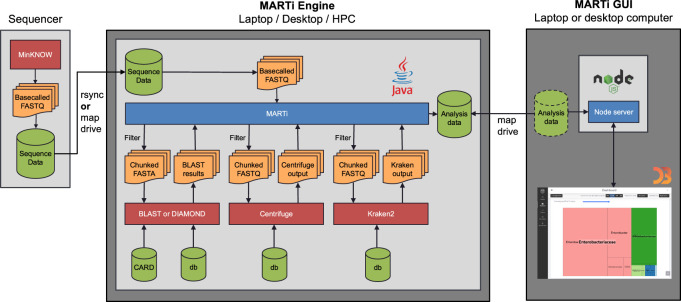
MARTi software architecture. MARTi consists of two main components: the MARTi Engine, a Java backend that performs the analysis of the sequencing data; and the MARTi GUI, a browser-based graphical user interface for visualizing, exploring, and comparing results.

MARTi consists of two main components: the MARTi Engine, a Java backend that performs the analysis of the sequencing data; and the MARTi GUI, an easy-to-use browser-based graphical user interface for visualizing, exploring, and comparing results (Fig. 1). The modularity of MARTi allows it to be configured in different ways depending on the needs of the experiment and the computational equipment available.

In the local configuration, both the Engine and GUI are installed on a single device. This configuration does not rely on any external computing resources and is suitable for in situ analysis. A nanopore sequencing device, such as a MinION or GridION, generates batches of base-called reads that are accessible to the MARTi computer either by mapping the sequencer's drive or via the *rsync* utility. The MARTi Engine, initiated through the command line or via the MARTi GUI, analyzes new data as it becomes available from the sequencer. The GUI server is run on the same computer and provides analysis results to any connected web browser, which could be a browser on the same computer or any other device, including tablets, on the same network.

In the HPC configuration, the MARTi Engine runs on an HPC or separate server, whereas MARTi GUI resides elsewhere, for example, on a laptop/desktop (Fig. 1). MARTi supports job scheduling via Simple Linux Utility for Resource Management (SLURM) on the HPC or its own process-based job scheduling (when SLURM is not available). This approach allows greater parallelization of analysis processes, facilitating queries against larger databases (including NCBI's nucleotide database), and enabling analysis of multiple runs simultaneously. The Engine analyzes the data on the HPC or separate server and generates output files for the front end. The MARTi GUI's server is run on a desktop/laptop that has access to the network location containing the output files.

### MARTi Engine

The MARTi Engine carries out the following main processes: prefiltering (to remove low-quality and short reads); classification (assigning reads to taxa); AMR analysis (an optional AMR gene detection step); and generating (writing output and analysis files for downstream analysis, including those needed by the GUI).

#### Prefiltering

Basecalled reads first pass through a prefilter that removes low-quality or short reads based on user-set thresholds. The default minimum length is 500 bp to remove short reads that have low taxonomic discriminatory power. The default minimum mean quality score is 9, equal to the pass read cutoff of ONT's High accuracy (HAC) basecalling model. Reads that pass prefiltering are batched into chunks of a specified size for further analysis. The division of reads into chunks permits the parallelization of the later stages.

#### Classification

The three main ways by which MARTi taxonomically classifies reads are BLAST (Camacho et al. 2009) (followed by its own Lowest Common Ancestor (LCA) algorithm using the NCBI taxonomy; see Methods), Centrifuge (Kim et al. 2016), and Kraken2 (Wood et al. 2019). MegaBlast is the default BLAST algorithm for nucleotide-to-nucleotide comparison within MARTi, but other options are supported via the *blastn -task* option. MARTi also supports translated nucleotide-to-protein comparison (with *blastx* and DIAMOND) and translated nucleotide-to-translated nucleotide (with *tblastx*). MARTi requires users to supply their own prebuilt reference databases that are locally accessible to the MARTi Engine at runtime; MARTi does not manage the download or construction of databases.

#### AMR analysis

If specified in the configuration file, the MARTi Engine will also BLAST the filtered reads to CARD for AMR gene identification. CARD's metadata is also used to assign drug class, resistance mechanism, and other information to hits. The host species of an AMR gene hit can sometimes be identified using the taxonomic classification of flanking DNA sequences, a process known as walkout analysis (Leggett et al. 2020). If a read is not long enough to contain significant flanking regions, it is likely to have hits to multiple species and therefore be assigned to a higher taxonomic level by the LCA algorithm. Similarly, AMR genes located on plasmids can pose a challenge as the flanking regions can often have ambiguous taxonomic hits.

#### Generating output

The Engine writes out analysis data and output files, including those required for the MARTi GUI to function. To reduce disk usage, the Engine will compress large intermediate files such as the BLAST output and chunked read files using gzip.

### MARTi GUI

**Figure 2.**
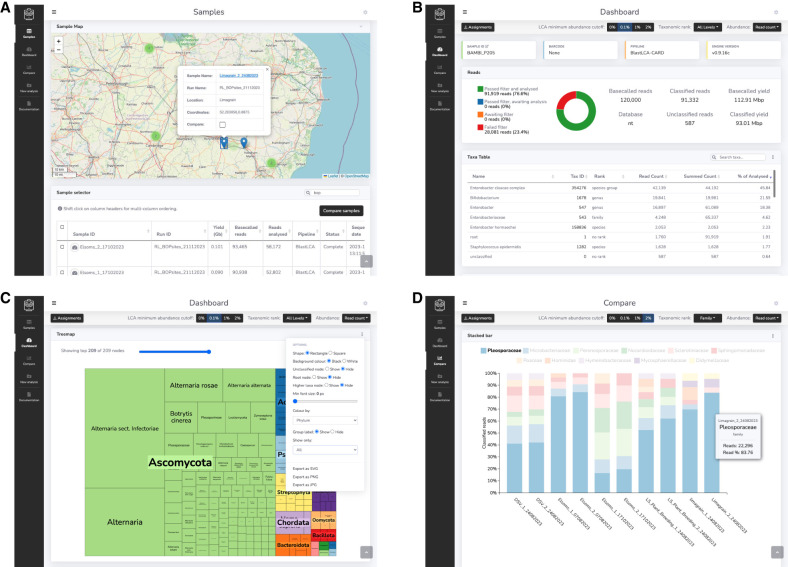
The MARTi GUI. (*A*) The Samples page is the landing page for the interface and allows users to view and load available samples. (*B*) The Dashboard is for viewing results of an individual sample. (*C*) The Dashboard displays analysis results using various tables and interactive plots that update in real time as updates become available. Each plot has an options menu (shown here on the treemap plot) for plot customization and export buttons. (*D*) The Compare page can be used to compare taxonomic composition and AMR hits between samples.

The MARTi GUI is a lightweight browser-based frontend that allows users to view and interact with results generated by the Engine without the need for in-depth bioinformatic knowledge or a strong understanding of the command line (Fig. 2). Crucially, the GUI will update as sequencing progresses and new data becomes available; assuming sufficient computer resources are available, this will appear in near real-time. The GUI has four pages: Samples, for selecting and loading available sample results; Dashboard, for viewing metrics and real-time analysis results of a single sample; Compare, for sample comparison; and New analysis, which allows users to configure and start a local MARTi analysis from the MARTi GUI. All plots on the GUI have options for customization, update automatically as new data is generated by the Engine, and can be exported as vector (e.g., for papers or presentations) or raster images. Plots on the Dashboard and Compare pages can be displayed at different taxonomic ranks—for example, family, genus, species—at four different LCA minimum abundance cutoffs (0%, 0.1%, 1%, and 2%, where reads from taxa with classified read proportions under the threshold are bumped up the tree until they are in a taxonomic bin that satisfies the minimum) and abundance can be based on read counts or base pair yield.

#### Samples

The main purpose of the Samples page is to allow users to select and load available samples into Dashboard and Compare analysis modes. However, it also has a data export panel and a sample map (Fig. 2A). The export panel enables users to export selected sample data in the format of their choice for downstream analysis, including formats accepted by other metagenomic tools. The map view plots samples with location metadata on a world map and can be used directly for sample selection. The markers displayed on the map reflect sample table filtering.

#### Dashboard

Taxonomic classifications, AMR hits, and other sample information for an individual sample can be viewed on the Dashboard page (Fig. 2B). Taxonomic results are displayed in a table and plotted as a donut, taxonomic tree, treemap, and a Sankey diagram (Fig. 2C), the latter similar in style to the one in Pavian, an interactive metagenomics visualization tool (Breitwieser and Salzberg 2020). The treemap provides a compact nested overview of relative abundances, whereas the Sankey visualizes flows from higher to lower taxonomic ranks, making dominant branches easier to identify. Together, these plots can help the user understand the taxonomic structure of complex communities. The page also features an accumulation curve that shows the number of taxa identified at the selected taxonomic rank over reads analyzed. If AMR analysis was carried out, the page will also feature a table of AMR hits, a host organism donut plot, and a donut plot of AMR genes, resistance mechanisms, and drug classes associated with species.

#### Compare

This page facilitates comparison of taxonomic composition and AMR presence (Fig. 2D). This page features a stacked bar chart, multidonut plot, combined taxonomic tree, taxonomic heat map, accumulation plot, and AMR heat map plot.

#### New analysis

The MARTi Engine requires a configuration file for each analysis, providing all the details for the analysis to be performed. A template configuration can be generated by the MARTi Engine using the *-writeconfig* flag. Alternatively, the New analysis page can be used to initiate local analysis or to generate a configuration file that can be downloaded and used elsewhere. It is not possible to initiate new runs on a remote HPC using the GUI, as varying HPC security and configuration approaches make it impossible to create a one-size-fits-all solution. Therefore, a command line option allows new analyses to be initiated in an HPC environment.

### Taxonomic classification pipeline evaluation

We evaluated the performance of 17 different classification pipelines (Table 1) using an in silico mock microbial community consisting of 100 k simulated reads with a read length N50 of ∼3.6 kb (Supplemental Table S1). We used three tools to run the pipelines, MARTi, EPI2ME agent, and EPI2ME (formerly EPI2ME Labs). For each of the MARTi pipelines, we compared the effect of setting LCA minimum abundance filters to 0%, 0.1%, and 1%. Across the experiments, three different read classification methods were deployed, Centrifuge, Kraken2, and MegaBLAST followed by a LCA algorithm. The intention of this exercise was not to evaluate the underlying classification algorithms (comprehensive benchmarking can be found in Pearman et al. 2020; Portik et al. 2022; Marić et al. 2024; Van Uffelen et al. 2024) but to compare their implementations within MARTi against those in the EPI2ME tools and to quantify the impact of MARTi-specific features, particularly its configurable LCA algorithm and minimum-abundance filtering, on read classification, taxon detection, and relative-abundance estimates.

**Table 1.** Taxonomic classification pipelines applied to the simulated mock community

Pipeline	Platform	Classification	Database
MARTi-BLAST-nt-0.0	MARTi	MegaBLAST	NCBI Nucleotide (03-2023)
MARTi-BLAST-nt-0.1	MARTi	MegaBLAST	NCBI Nucleotide (03-2023)
MARTi-BLAST-nt-1.0	MARTi	MegaBLAST	NCBI Nucleotide (03-2023)
MARTi-BLAST-meta-0.0	MARTi	MegaBLAST	Metagenomic (11-2023)
MARTi-BLAST-meta-0.1	MARTi	MegaBLAST	Metagenomic (11-2023)
MARTi-BLAST-meta-1.0	MARTi	MegaBLAST	Metagenomic (11-2023)
MARTi-Centrifuge-meta-0.0	MARTi	Centrifuge	Metagenomic (11-2023)
MARTi-Centrifuge-meta-0.1	MARTi	Centrifuge	Metagenomic (11-2023)
MARTi-Centrifuge-meta-1.0	MARTi	Centrifuge	Metagenomic (11-2023)
EPI2ME-Centrifuge-meta-0.0	EPI2ME Agent	Centrifuge	Metagenomic (06-2021)
MARTi-Kraken2-pluspf8-0.0	MARTi	Kraken2	PlusPF-8 (03-2023)
MARTi-Kraken2-pluspf8-0.1	MARTi	Kraken2	PlusPF-8 (03-2023)
MARTi-Kraken2-pluspf8-1.0	MARTi	Kraken2	PlusPF-8 (03-2023)
EPI2ME-Kraken2-pluspf8-0.0	EPI2ME	Kraken2	PlusPF-8 (03-2023)
MARTi-BLAST-pluspf8-0.0	MARTi	MegaBLAST	PlusPF-8 (03-2023)
MARTi-BLAST-pluspf8-0.1	MARTi	MegaBLAST	PlusPF-8 (03-2023)
MARTi-BLAST-pluspf8-1.0	MARTi	MegaBLAST	PlusPF-8 (03-2023)

### Read classification

**Figure 3.**
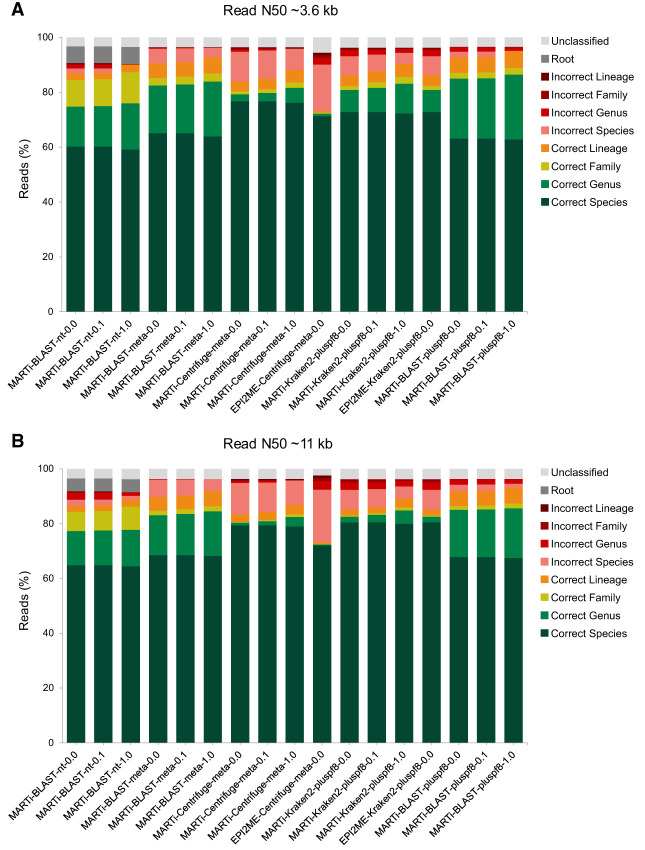
Read classifications for different pipelines using a small mock microbial community consisting of 100 k simulated reads. (*A*) Read classifications using trimmed down reads with a read N50 of ∼3.6 kb. (*B*) Read classification results using the longer reads, read N50 of ∼11 kb.

To evaluate the read-level classification performance of each pipeline, we compared the taxonomic classification of each read with its known source (Fig. 3A,B). We assessed classification performance using our simulated metagenomic data set (proportionally shortened to create a more realistic ∼3.6-kb N50 read length) and the full-length simulated data set it had been generated from (∼11-kb N50). We also calculated recall, precision, and F-scores, F_1_ and F_0.5_, at genus and species levels (Supplemental Table S2). We define read classification recall as the number of reads correctly classified divided by the total number of reads (100 k in this case). Precision was calculated as the number of correctly classified reads divided by the number of assigned reads at a particular taxonomic level and below (i.e., unassigned and reads assigned above a given taxonomic rank are not counted). F-scores were used to summarize the precision and recall scores for each pipeline. F_1_ is the harmonic mean of precision and recall, weighting them equally. F_0.5_ gives more weight to precision, favoring pipelines that minimize false-positive classifications.

The proportion of reads classified was high across all pipelines, ranging from 94.43% to 96.78% for the shorter read set, and from 96.21% to 97.60% for the longer reads. For both read sets, the EPI2ME-Centrifuge-meta-0.0 pipeline had the highest proportion of incorrectly assigned reads (22.53% and 25.09% for shorter and longer reads, respectively), the lowest precision at both species and genus level, and the lowest recall at genus level. Conversely, the MARTi-BLAST-nt-1.0 pipeline had the lowest proportion of incorrectly assigned reads (shorter read set: 0.17%, longer read set: 2.69%), had perfect precision at both species and genus level for the shorter read set, and the highest precision at species level for the longer read set. However, the MARTi-BLAST-nt-1.0 method also had the lowest species-level recall for both data sets (shorter read set: 0.59, longer read set: 0.65), and the MARTi-BLAST-nt pipelines were the only methods that classified a considerable proportion of reads to the “Root” node (shorter read set: 6.17%, longer read set: 4.72%).

MARTi-Kraken2-pluspf8-0.0 and EPI2ME-Kraken2-pluspf8 produced identical results, as expected, given that we used the same classification database and version of Kraken2. With both data sets, we observed that Kraken2- and Centrifuge-based pipelines had higher species-level recall than BLAST-based pipelines. However, BLAST-based pipelines always had the highest species-level precision for each database used. Regardless of the classification method, implementing a minimum abundance cutoff within MARTi consistently increased the proportion of correctly assigned reads.

### Taxon detection

**Figure 4.**
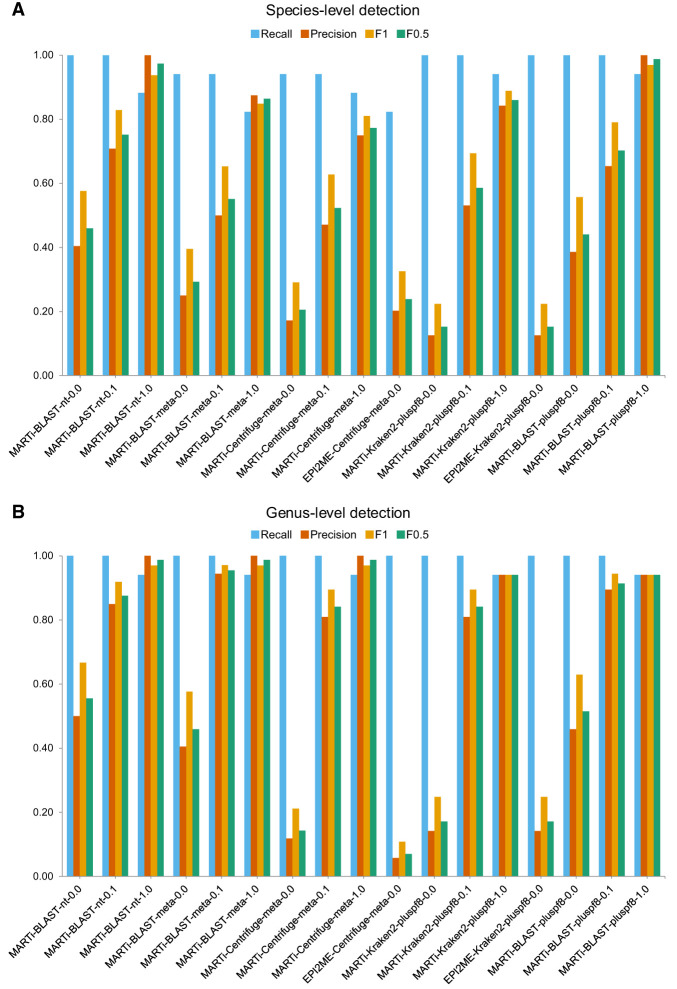
Taxa detection metrics (recall, precision, F1, and F0.5) for different pipelines using simulated reads (read length N50 ∼3.6 kb) from a small mock microbial community. (*A*) Species-level detection metrics. (*B*) Genus-level detection metrics.

The ability to accurately detect organisms present in the mock was evaluated across the different pipelines at both species and genus level using the shorter read set (Fig. 4A,B). At each level, we calculated recall, precision, and F-scores, F_1_ and F_0.5_ (Supplemental Table S3). Taxa detection recall was defined as the number of correctly identified taxa divided by the total taxa expected; thus, a score of 1 indicates all expected taxa were detected. Precision was calculated as the number of correctly identified taxa at a taxonomic level divided by the total number of taxa identified at that level (true positives + false positives).

At the species level, 7/17 of the methods successfully detected all 17 species present within the mock community. The MARTi-Blast-meta-1.0 and EPI2ME-Centrifuge-meta-0.0 pipelines had the lowest species-level recall, detecting 14 of the 17 species. MARTi-Centrifuge-meta-0.0 detected a greater number of expected species (16/17) than the EPI2ME-Centrifuge-meta-0.0 pipeline (14/17), likely due to its use of a more recent database. Kraken2-based pipelines without an LCA cutoff had the highest number of false-positive species detections. However, the majority of these false positives were supported by very few reads and applying a 1% minimum cutoff removed almost all of them, thereby increasing precision. MARTi-BLAST-nt-1.0 and MARTi-BLAST-pluspf8-1.0 achieved perfect precision scores, with no false-positive species detections, and the highest F_0.5_ scores (0.97 and 0.99, respectively).

Genus-level analysis generally yielded higher recall and precision compared to species-level analysis. At this level, 12 out of 17 methods successfully detected all 17 genera in the mock data set. Similar to species-level results, applying a minimum abundance cutoff at the genus level typically reduced recall while increasing precision. The five pipelines that detected 16 out of 17 genera all used a 1% minimum abundance cutoff and consistently missed the *Clostridium* genus, which had the lowest abundance at just 0.5%. The highest F_1_ and F_0.5_ scores were achieved by MARTi-BLAST-meta-0.1 and MARTi-BLAST-meta-1.0, respectively.

### Relative abundance estimation

**Figure 5.**
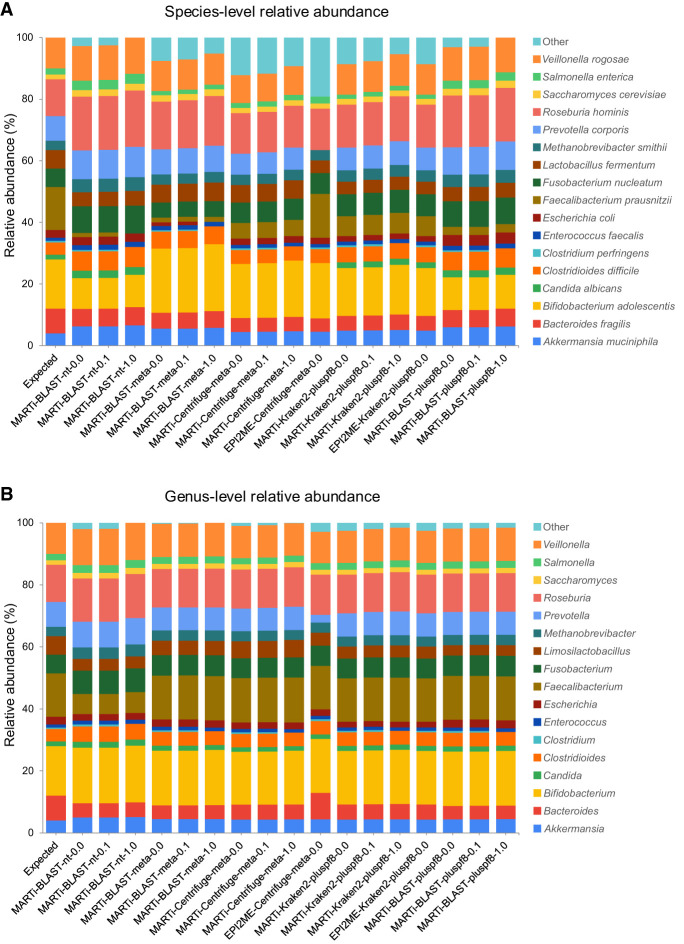
Relative abundance estimates for different classification pipelines using simulated reads from a small mock microbial community at (*A*) species-level, and (*B*) genus-level. The first bar represents the expected abundances of each taxon in the mix based on read counts. False-positive classifications are grouped into “Other.”

We assessed each pipeline's ability to estimate relative taxonomic abundance based on read counts. The resulting communities were compared using the known composition of the simulated data set (Fig. 5A,B) and Bray-Curtis dissimilarities (Supplemental Table S4). At the species level, abundance estimates by the Kraken2-based methods were the most accurate (based on Bray-Curtis dissimilarity values), followed closely by the MARTi-Centrifuge-meta pipelines. EPI2ME-Centrifuge-meta had the highest dissimilarity and therefore was considered to have the least accurate species-level abundance estimation. The second most relatively abundant species in the simulated data set, *Faecalibacterium prausnitzii* (14% of the reads), was generally underrepresented by most methods, with values ranging from 0% to ∼6.7%, with the exception of EPI2ME-Centrifuge-meta, which reported a more accurate relative abundance of ∼14.2%.

For every pipeline, relative abundance profiles were more accurate at the genus level than at the species level. At the genus level, MARTi-Centrifuge-meta pipelines had the lowest dissimilarity scores and appeared most like the expected profile. MARTi-BLAST-nt pipelines had the highest dissimilarity scores, most likely due to their underestimation of the *Faecalibacterium* genus (expected: 14%, MARTi-BLAST-nt pipelines: 6.53%–6.70%, all other pipelines: 13.97%–14.48%).

### Local configuration analysis rate

We compared the analysis rate of the three main classification methods available within MARTi by analyzing 100 k simulated mock microbial reads (∼1 Gbp of data) on two systems: a MacBook Pro and a Dell Precision 7920 Tower Workstation (Table 2). In the MARTi configuration files, for each method, we set the maximum number of parallel MARTi jobs and specified the number of threads each classification tool could use to optimize the tools’ use of computational resources.

**Table 2.** Analysis rate and memory use for the main classification pipelines within MARTi running on a MacBook Pro and Dell server

Pipeline	Device	Jobs	Threads	Peak memory (MB)	Execution time (s)	Reads per min (RPM)
MARTi-BLAST-meta	MacBook	8	1	65,526	47,457	126.43
MARTi-Centrifuge-prok	MacBook	2	8	41,958	224	26,785.71
MARTi-Kraken2-pluspf8	MacBook	2	8	48,142	204	29,411.76
MARTi-BLAST-meta	Dell Tower	20	2	126,041	18,616	322.30
MARTi-Centrifuge-meta	Dell Tower	1	40	191,418	2778	2159.83
MARTi-Kraken2-meta	Dell Tower	1	40	156,038	1264	4746.84
MARTi-Centrifuge-prok	Dell Tower	40	1	191,269	328	18,292.68
MARTi-Kraken2-pluspf8	Dell Tower	40	1	123,588	271	22,140.22

On the MacBook Pro, Kraken2 was the fastest, with a total wall-clock analysis time of 204 sec, classifying around 29,412 reads per minute (rpm). This was closely followed by Centrifuge, which took 224 sec, classifying at 26,786 rpm. BLAST was by far the slowest, with a total analysis time of 49,895 sec (i.e., ∼14 h or overnight), analyzing at a rate of 120 rpm.

Kraken2 remained the fastest classifier on the Dell Tower. BLAST performance improved substantially on the Tower, with the rpm increasing from ∼126 to ∼322, underscoring the importance of hardware when selecting classification methods.

### Profiling of preterm infant microbiota

**Figure 6.**
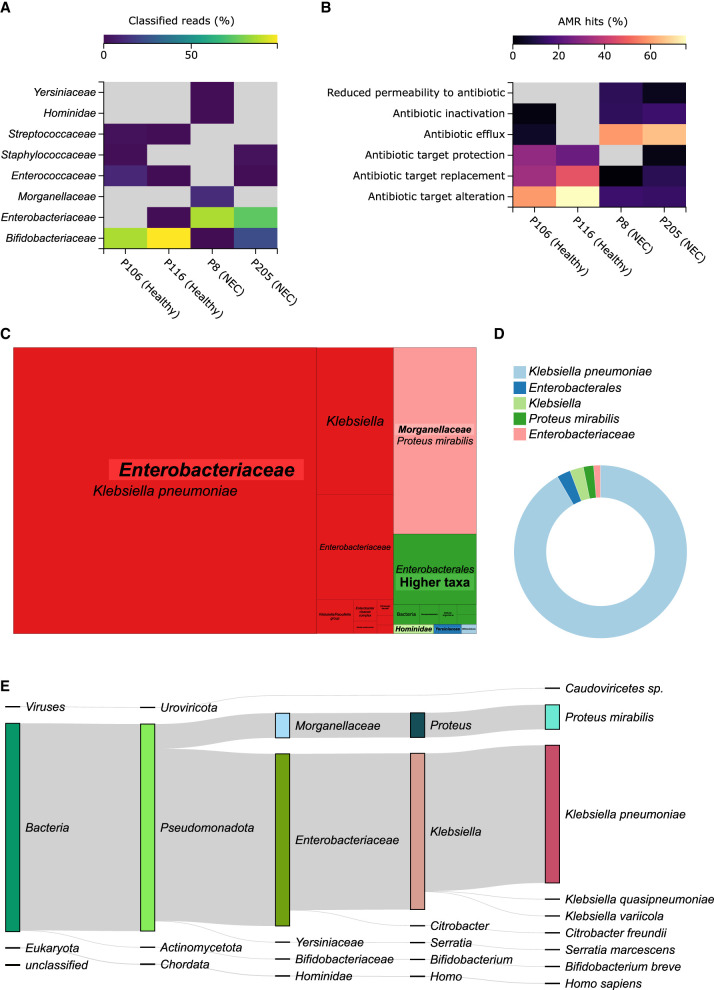
Selected MARTi GUI plots from the re-analysis of four published preterm infant microbiomes. Two of the samples were from healthy individuals (P106 and P116) and the other two from preterms clinically diagnosed with NEC (P205 and P8). (*A*) Family-level taxonomic classification heat map. (*B*) Resistance mechanism composition of the AMR hits within each sample. (*C*) Treemap of classified reads for P8 (an NEC sample) showing all taxonomic levels grouped by family. (*D*) Donut plot displaying the proportions of AMR hits for P8 that have been associated with taxa by “walkout” analysis. A 0.1% minimum LCA abundance threshold was used for all the taxonomic classification plots. (*E*) Sankey diagram of classified reads for P8 (an NEC sample) illustrating read assignment flows from higher to lower ranks. Plots were exported from the MARTi GUI as SVGs and arranged with Inkscape, where species and genera names were also italicized.

To demonstrate the application of MARTi on real data, we reanalyzed four published preterm infant microbiomes (Leggett et al. 2020) using the MARTi-BLAST-nt pipeline. Two of the samples were from healthy individuals (P106 and P116), and the other two from preterms clinically diagnosed with necrotizing enterocolitis (NEC) (P205 and P8). We then used MARTi GUI to explore the results (Fig. 6). In accordance with the original study, we found the microbiota of healthy samples was dominated by *Bifidobacteriaceae*, whereas that of NEC patients was dominated by *Enterobacteriaceae* (Fig. 6A). The most abundant taxa were consistent and displayed similar relative abundances to those in the original study. In sample P205 (NEC), the dominant taxon was the *Enterobacter cloacae* complex, and in sample P8 (NEC), it was *Klebsiella pneumoniae* (Fig. 6C,E).

We also ran MARTi's AMR analysis on the microbiome data sets. We observed distinct differences in AMR resistance mechanism profiles between healthy and NEC samples, with a significant portion of the AMR hits in NEC individuals associated with antibiotic efflux and inactivation mechanisms that were almost entirely absent from the healthy samples (Fig. 6B). Most of the AMR hits within sample P205 (NEC) were associated with *E. cloacae*, whereas within sample P8 (NEC), they were assigned to *K. pneumoniae* (Fig. 6D). We used a more recent version of the CARD database for our reanalysis with MARTi, and as a result, we identified many antimicrobial resistance ontologies (AROs) that were not present in the database used for the original analysis. Notably, 15 of the 32 AROs identified by MARTi in P8 (NEC), including the top four AROs by read count, were absent from the database used in the original analysis.

### Zymo mock community composition

To further benchmark MARTi using real-world data, we analyzed the ZymoBIOMICS Even Mock Community (D6300) nanopore data set published by Nicholls et al. (2019) using BLAST with NCBI's core nt database. MARTi was able to detect all 10 expected species with no false positives (with a 0.1% minimum abundance LCA threshold applied). Relative abundances were generally concordant with both the expected composition and previously published results (Supplemental Table S6). Two of the organisms, *Limosilactobacillus fermentum* and *Bacillus spizizenii*, were reported under their earlier taxonomic names in the original study (*Lactobacillus fermentum* and *Bacillus subtilis*, respectively) but were reported by MARTi using current nomenclature.

## Discussion

ONT's long-read sequencing platforms are the first to enable progressive real-time data analysis, with the potential to revolutionize metagenomics by improving classification accuracy and assembly quality, reducing time to results, and enabling new ways of working, such as bringing the lab to the sample. However, the full potential of nanopore sequencing remains largely unrealized due to the lack of suitable analysis tools and pipelines. In this manuscript, we present MARTi, an open-source software tool that enables real-time analysis and visualization of metagenomic sequencing data. MARTi provides a rapid, lightweight web interface that allows users to view community composition and identify antimicrobial resistance genes in real-time.

To our knowledge, MARTi is the first open-source tool to offer live data visualization, comparison of multiple samples, AMR profiling, taxonomic classification with multiple pipelines, and a user-friendly interface requiring no command-line expertise. These features make MARTi especially suited for applied contexts such as pathogen surveillance, point-of-care diagnostics, and biodiversity monitoring where time-to-result and ease of use are critical. MARTi's real-time visualization of taxonomic and AMR classification results enable users to monitor results as sequencing progresses. MARTi GUI plots such as the accumulation curve, which shows the number of taxa identified at the selected taxonomic rank over reads analyzed, can help users assess whether sufficient data has been collected to answer a specific question, such as detecting pathogens or community shifts. This could enable informed decisions about whether to stop a run early, thus saving time and resources. MARTi can just as easily be run on historical data or after a run has completed, serving as a user-friendly, feature-rich first line analysis tool.

Whereas MARTi relies on established classification tools, its novelty lies in integrating these within an open-source, real-time, and highly configurable platform that operates both offline and on HPC systems. MARTi represents a new way to access well-tested algorithms and to visualize metagenomic results during analysis through an intuitive, browser-based interface. Importantly, MARTi implements additional features such as prefiltering, read chunking, a configurable Lowest Common Ancestor algorithm, and LCA minimum abundance cutoff levels. Benchmarking MARTi's performance against other tools that use the same underlying classification methods is, therefore, essential, as it demonstrates that these enhancements maintain and, in some cases, improve performance while offering greater flexibility and usability.

MARTi allows users to classify their reads in three main ways: BLAST, followed by a LCA algorithm; Centrifuge; and Kraken2. We have demonstrated and compared each of these methods using simulated gut microbiome reads, where the true origin of each read is known. We found that *k*-mer-based tools, Kraken2 and Centrifuge, had the highest species-level recall. This means that they correctly classified the greatest number of reads to the species level, beating BLAST-based pipelines using equivalent databases. However, the *k*-mer-based methods also incorrectly classified the greatest number of reads at species level, leading to lower precision than BLAST-based methods. The ideal classification pipeline would achieve both high precision and recall, but in practice, classification tools often exhibit a trade-off between these metrics (Portik et al. 2022). For many metagenomic applications, high precision at the expense of recall, as seen with BLAST-based methods, will be more desirable in order to minimize false-positive classifications.

Whereas DIAMOND is also supported in MARTi for translated nucleotide-to-protein comparisons, particularly for protein-level or functional classification tasks, we did not benchmark it in this study. Our evaluation focused on nucleotide-based classifiers (BLAST, Kraken2, Centrifuge) to reflect common taxonomic use cases in long-read metagenomics. Future work may explore the utility of DIAMOND within MARTi for metagenomic functional profiling.

Read classification recall was improved for every method when using the longer simulated reads—∼11-kb read length N50—with the biggest increase seen in Kraken2-based pipelines at the species level. However, precision was usually slightly reduced using longer reads, with the most notable exception being the Kraken2 pipelines, which saw increases in species-level precision. Similar results have been reported by Pearman et al. (2020), who showed that BLAST and Kraken2 recall was improved by the use of longer reads, but precision only improved for Kraken2.

Within the MARTi GUI, users can view taxonomic classification results with different minimum abundance cutoff values (0%, 0.1%, 1%, and 2%). These filter out low-abundance taxa by promoting them to higher taxonomic ranks. This feature helps reduce noise in community profiles and improves interpretability. We demonstrated that applying a cutoff value can improve these classification tools’ overall taxonomic detection results, especially the *k*-mer-based tools, Kraken2 and Centrifuge, that had higher numbers of false positives with no filtering. In concurrence with Portik et al. (2022), we observed that the application of a minimum abundance threshold can reduce the number of false-positive detections, increasing precision, but at the cost of increased false negatives, reducing recall, especially at more stringent threshold values.

We estimated relative taxonomic abundances for each of the analysis pipelines applied to the simulated mock gut microbiome. Abundance was estimated using cumulative read counts, the sum of the reads assigned to a taxon and below, and then we calculated Bray-Curtis dissimilarities to compare each of the estimated mock community compositions to the known composition. Kraken2-based methods produced species-level abundance profiles most similar to the expected profile, whereas MARTi-Centrifuge-meta pipelines exhibited the lowest dissimilarity scores at the genus level.

A potential limitation of using long-read count data for abundance estimation is variability in read lengths across different species within a mix, which could lead to skewed abundance estimates. To account for this, we have added an option within the MARTi GUI for users to switch between read-count-based abundance and abundance based on cumulative assigned base pairs. This feature aligns with findings by Marić et al. (2024), who demonstrated that incorporating read length information can improve abundance estimation accuracy for ONT data sets.

For real-time metagenomic applications, analysis rate should be greater than or equal to sequencing rate to keep up. We demonstrated that MARTi can analyze ∼1 Gbp every 3.5 min when using Kraken2 for classification on a MacBook Pro (Table 2). At this rate, MARTi can analyze the data from a 72-h MinION run (producing 48 Gbp theoretical max output) in under 3 h on a laptop. The analysis rates we observed for Kraken2 and Centrifuge were similar to those reported by Marić et al. (2024).

Whereas BLAST is often considered the gold standard for sequence comparison, it is orders of magnitude slower than modern *k*-mer-based tools such as Kraken2 and Centrifuge. However, our benchmarking shows that a high-spec workstation, such as a Dell Precision 7920, can substantially improve BLAST performance compared to a MacBook Pro, primarily due to increased available memory and parallelization. We therefore recommend that users who wish to run BLAST-based classification with MARTi do so on an HPC or a powerful workstation to ensure practical runtimes.

As highlighted by recent benchmarking studies, there is no single “best” classification tool for all scenarios (Portik et al. 2022; Van Uffelen et al. 2024). Different tools offer trade-offs between speed, sensitivity, and precision. The optimal choice depends on the research question and sample complexity. Based on recent benchmarking studies and our own results, we suggest using Kraken2 for real-time analyses where speed is a priority and sample complexity is low or users are identifying presence/absence of a species, such as rapid pathogen detection. Although often outperformed by Kraken2, Centrifuge shows strong genus-level performance in our tests and has exceeded Kraken2 in specific scenarios, including simple microbial communities and simulations of ancient DNA (Leidenfrost et al. 2020; Arizmendi Cárdenas et al. 2022). For studies where higher classification precision is essential, such as microbiome studies of complex communities with closely related species, we recommend using BLAST with an appropriate LCA threshold, although this is best run on an HPC or powerful workstation due to its computational demands.

We re-analyzed published infant microbiome data sets with MARTi using MegaBlast against the nt database for classification. The main taxonomic findings were the same, with some differences in the less abundant taxa. This is not surprising considering a similar BLAST-based approach was taken in the original study, with differences in results largely due to growth in the nt database and how MARTi's LCA algorithm interpreted the BLAST output. We also found that up to ∼40% of AMR genes identified in the re-analyzed samples were not previously found due to being absent from the database used in the original study. These results highlight the impact of parameter and database choice on metagenomic classification. One feature of MARTi that is not present in EPI2ME is the sample comparison mode, making it easy to compare the taxonomic assignments and AMR hits across samples. This has obvious uses for comparing biological communities between different sites, conditions, and time points. However, the compare mode is also useful for assessing the effect of database and parameter choice on the same data set.

We further benchmarked MARTi using published ZymoBIOMICS mock community data. MARTi detected all expected taxa with no false-positive species when using a 0.1% minimum abundance threshold. The resulting abundance profiles resembled the expected composition and were similar to those reported by Nicholls et al. (2019). Discrepancies in relative abundances for some taxa, such as *Escherichia coli* and *Listeria monocytogenes*, appear to result from a higher fraction of reads being assigned to further up the taxonomic tree. This likely reflects the greater sequence similarity of these organisms to closely related taxa in the database and the more conservative nature of the BLAST-LCA pipeline. Notably, unlike the Nicholls et al. (2019) analysis, which mapped reads directly to genome assemblies, MARTi used BLAST searches against the full NCBI core nt database.

A key strength of MARTi is its support for user-provided reference databases across all supported classifiers. This enables researchers to tailor the underlying reference database to suit specific sample types or research questions. In addition, MARTi also supports taxon exclusion during classification where this is provided by the underlying tool. Currently, BLAST and Centrifuge support options that allow users to exclude specific taxa from the alignment by providing a taxonomic ID filter list. This functionality is particularly useful in studies where host contamination is a concern, or when working with comprehensive databases like NCBI nt and wishing to exclude synthetic sequences and low-quality entries that could reduce classification accuracy.

MARTi represents a significant advance in real-time metagenomics, addressing the need for a flexible, open-source, and offline analysis tool. Through comprehensive evaluation using simulated and real-world data sets, MARTi has demonstrated robust performance in read classification, taxon detection, and relative abundance estimation, leveraging classification tools BLAST, Centrifuge, and Kraken2. Although initially developed for long-read metagenomics, MARTi can be applied to short reads and metabarcoding projects with some parameter changes. The integration of an intuitive, browser-based GUI facilitates accessibility and usability, making metagenomic analysis more attainable for diverse research settings. Furthermore, MARTi's capability to operate in different configurations ensures scalability and adaptability to varying computational resources, making it a useful tool for real-time in-field metagenomic studies and larger scale postrun analysis. Future enhancements and community contributions will likely expand MARTi's functionality, further solidifying its role in metagenomic data analysis.

## Methods

### Lowest Common Ancestor algorithm

When classifying reads using BLAST (nucleotide or protein) or DIAMOND (a BLAST-like protein alignment tool), MARTi implements a Lowest Common Ancestor algorithm (see below) to assign reads to taxa based on the alignment results. This algorithm assigns reads to the lowest taxonomic level consistent with “good” hits. The definition of good is configurable and depends on the BLAST bit score, length of match, percent identity of the match, and the maximum number of hits to consider. MARTi implements a Lowest Common Ancestor algorithm as follows:

1. Reads are BLASTed against a user-defined database. This may be, for example, the whole of NCBI nt, a bacteria subset, RefSeq genomes, proteins such as NCBI's nr, or a custom database. This results in a set of between 0 and many hits for each read.
2. For a given read, the set of “good hits” is identified by finding the highest scoring hit (according to the BLAST bitscore), then finding all hits with a score within 90% (default value, but configurable) up to an optional limit (default 100 hits, but configurable). Note, if the number of hits considered is limited, there is a risk that the first X hits may not contain the best hit; the larger this number, the lower the risk of misclassification.
3. For each good hit, the taxonomic path is determined by referring to the NCBI taxonomy. For example:
  "root, cellular organisms, Bacteria, Proteobacteria, Gammaproteobacteria, Enterobacterales, Enterobacteriaceae, *Klebsiella*, *Klebsiella pneumoniae*"

The taxonomic paths for all good hits are compared to determine the common ancestor. This involves starting at the root node and working downwards, comparing nodes (first “root”, then “cellular organisms”, then “bacteria,” etc.) until paths diverge. The last node in common before paths diverge is the lowest common ancestor and the read is assigned to this taxon.

The MARTi Engine generates a sample's taxonomic tree using four LCA minimum abundance cutoff points (0%, 0.1%, 1%, and 2%), calculated based on both read counts and base pair yield. When a cutoff is applied, reads assigned to taxa that fall below the selected threshold are not discarded but instead reassigned by bumping them up the tree to the nearest ancestral taxon that passes the threshold. This ensures that all classified reads are retained in the final output, while removing low-abundance taxa. To support fast, interactive filtering in the MARTi GUI, these threshold-specific trees are built by the MARTi Engine after each new chunk of reads is analyzed.

### Mock microbial community read simulation

We generated a small mock microbial community data set consisting of 100 k simulated nanopore reads to evaluate the classification pipelines available within MARTi (Supplemental Table S1). Long reads were simulated from 17 RefSeq genomes (15 prokaryotes and 2 eukaryotes) using NanoSim v3.1.0 (Yang et al. 2017), subsampled, then combined and randomly shuffled. The original simulated data set has a total of 1,041,760,674 base pairs (2.09 GB of FASTQ files) and an N50 read length of 10,952 bp. To generate a more realistic metagenomic data set, we reduced each read to one third of its original length, resulting in a read length N50 of 3614 bp.

### Reference databases

For classification benchmarking, we chose commonly used databases for each of the classification tools available through MARTi. We aimed to use equivalent reference databases wherever possible to enable fairer comparisons across pipelines. A summary of the databases used, including their content, number of taxonomic IDs, and disk size, is provided in Supplemental Table S5.

The database used for all Kraken2 pipelines, in MARTi and EPI2ME, was PlusPF-8 (March 2023, downloaded from https://benlangmead.github.io/aws-indexes/k2), a prebuilt Kraken2 index containing references for archaea, bacteria, viral, plasmid, human, UniVec_Core, protozoa, and fungi. The EPI2ME-Kraken2 pipeline classified the simulated reads to this database with the wf-metagenomics workflow (v2.8.0).

EPI2ME agent's WIMP workflow (v2023.06.13-1865548) uses Centrifuge to classify reads to a metagenomic database consisting of archaea, bacteria, viral, fungal, and human RefSeq genomes (downloaded June 2021). As we could not access the exact database version used by ONT, we downloaded and built a more recent equivalent (Nov 2023) for the MARTi-Centrifuge-meta pipeline with Centrifuge v1.0.3. We refer to this database as “Metagenomic (11-2023).”

The database was downloaded with the following commands:

centrifuge-download -o taxonomy taxonomy

centrifuge-download -o library -m -d “archaea,bacteria,viral,fungi” refseq > seqid2taxid.map

The human genome was added as follows:

centrifuge-download -o library -d “vertebrate_mammalian” -a “Chromosome” -t 9606 -c “reference genome” refseq >> seqid2taxid.map

The sequences were combined:

find library -name “*.fna” -exec cat {| >> input-sequences.fna \;

Then, the centrifuge index was built with the following command:

centrifuge-build -p 100 ‐‐conversion-table seqid2taxid.map ‐‐taxonomy-tree taxonomy/nodes.dmp ‐‐name-table taxonomy/names.dmp input-sequences.fna metagenome

For BLAST, which is not available in EPI2ME, we used several different databases. For the MARTi-BLAST-nt pipeline, we used NCBI's Nucleotide database (nt; https://www.ncbi.nlm.nih.gov/nucleotide/), a very large and comprehensive collection of nucleotide sequences. The nt database was downloaded as a prebuilt BLAST database from the NCBI FTP site (https://ftp.ncbi.nlm.nih.gov/blast/db). We used the March 2023 version for taxonomic classification pipeline evaluation and March 2024 version for preterm infant microbiota analysis.

For the MARTi-BLAST-pluspf8 pipeline, we constructed a BLAST database using the same sequences found in the Kraken2 pluspf8 database (March 2023). The following command was used to make the database:

makeblastdb -in sequences.fasta -parse_seqids -blastdb_version 5 -title “k2_pluspf_08gb” -dbtype nucl -taxid_map taxid_map.txt

We also built a BLAST database from the sequences used in the Centrifuge database (Nov 2023) for a more direct comparison of classification performance between Centrifuge (MARTi-Centrifuge-meta) and BLAST (MARTi-BLAST-meta) using the following command:

makeblastdb -in input-sequences.fna -parse_seqids -blastdb_version 5 -title “refseq_metagenomics” -dbtype nucl -taxid_map taxid_map.txt

### Pipeline read classification

We calculated the recall and precision at genus and species levels for each of the classification pipelines. We define read classification recall as the number of reads correctly classified divided by the total number of reads (100 k, in this case). Precision was calculated as the number of correctly classified reads divided by the number of assigned reads at a particular taxonomic level and below (i.e., unassigned and reads assigned above a given taxonomic rank are not counted). We also calculated *F*-scores as a way of summarizing the recall and precision information. The F_1_ score is the harmonic mean of precision and recall, providing an equally weighted view of the recall and precision. When calculating the F_0.5_ score, we emphasized precision, placing more importance on minimizing false-positive classifications. The highest value for either F-score is 1, indicating perfect precision and recall. The formulas for F_1_ and F_0.5_ are as follows:

$$F_{1}=(2\;*\;\mathrm{precision}\;*\;\mathrm{recall})/(\mathrm{precision}+\mathrm{recall}),$$

$$F_{0.5}=((1+0.5^{2})\;*\;\mathrm{precision}\;*\;\mathrm{recall})/((0.5^{2}\;*\;\mathrm{precision})+\mathrm{recall}).$$

### Taxa detection

We scored the presence/absence of taxa for each classification pipeline. We calculated recall, precision, and F-scores for taxa detection at genus and species levels. In this context, we define the taxa detection recall as the number of identified mock taxa divided by the expected number of taxa. Precision was calculated as the number of mock taxa identified divided by the total number of taxa detected at the species or genus level.

### Relative abundance estimation

Relative abundances were estimated for each of the classification pipelines at the species and genus level. For all pipelines, we estimated abundance using cumulative read counts for each taxon, which is the sum of the reads assigned to that taxon and below. The sum of the cumulative counts for false-positive taxa were grouped as “Other.” The counts were then normalized as percentages. To compare each of the estimated mock community compositions to the known composition, Bray-Curtis dissimilarity was calculated using the braycurtis function from the scipy.spatial.distance module in Python.

### Local configuration analysis rate

We analyzed the simulated mock microbial community data with MARTi running in local configuration on a MacBook Pro (2019, 8-core Intel Core i9-9880H @ 2.3 GHz, 64 GB DDR4 RAM) and a Dell Precision 7920 Tower (2x Intel Xeon Silver 4114 CPUs @ 2.20 GHz, 192 GB DDR4 RAM). The reads were classified with each of the three main methods available in MARTi: BLAST, Centrifuge, and Kraken2. For BLAST, we used the Metagenomic (11-2023) database downloaded with the centrifuge-download command (described earlier in Reference databases). A prebuilt index containing RefSeq prokaryotes was used for Centrifuge classification (April 2018, downloaded from https://benlangmead.github.io/aws-indexes/centrifuge). For the Kraken2 pipeline, we used the prebuilt PlusPF-8 index described previously. We also ran Kraken2 and Centrifuge on the Metagenomic (11-2023) database we used for BLAST to provide a more direct comparison across all classification tools.

### Profiling of preterm infant microbiota

To demonstrate MARTi on real data, we re-analyzed four published preterm infant microbiome data sets (Leggett et al. 2020), two from healthy individuals (P106 and P116), and two from preterms clinically diagnosed with NEC (P205 and P8). As described in the original paper, the sequence data is available from the European Nucleotide Archive (ENA; https://www.ebi.ac.uk/ena/browser/home) under accession PRJEB22207. We analyzed the first 120 k basecalled reads of P106, P116, and P205, and the first 100 k reads of P8 using MARTi v0.9.16. Reads passing MARTi's prefilter, minimum length 500 bp and minimum read quality score of 9, were taxonomically classified using the BLAST pipeline with NCBI's nt database (March 2024). Additionally, AMR gene analysis was carried out using the CARD v3.2.7 database. Taxonomic assignments and AMR analysis results were explored with the MARTi GUI.

### ZymoBIOMICS mock community analysis

We evaluated MARTi's classification performance with the ZymoBIOMICS Microbial Community DNA Standard (Even, D6300) GridION dataset published by Nicholls et al. (2019). FASTQ reads were downloaded from ENA (under accession number PRJEB29504) and processed using the MARTi BLAST classification pipeline with default parameters. The NCBI core nucleotide database (October 2024) was used as the reference, and results were filtered using a LCA minimum abundance threshold of 0.1%. Read assignments were aggregated at the species level, and relative abundances were calculated both as a percentage of total analyzed reads and as a percentage of all reads classified to species level. Abundance estimates were compared against expected values provided by the manufacturer and the previously published values derived from direct read mapping to genome assemblies.

### Software availability

The latest version of the MARTi software is available from GitHub (https://github.com/richardmleggett/MARTi) under the MIT License. A snapshot of the MARTi source code has also been included as Supplemental Code. Documentation can be found at https://marti.readthedocs.io/en/latest/. Additionally, an installation-free demo of the MARTi GUI is available at https://marti.cyverseuk.org/.

The simulated reads used in this study, including both the full-length and one-third length data sets, are available at Zenodo (https://doi.org/10.5281/zenodo.14260487).

## Supplemental Material

Supplement 1

Supplement 2

Supplement 3

Supplement 4

Supplement 5

Supplement 6

Supplement 7
