# Supplementary figures and images for: Real-time analysis and visualization of nanopore metagenomic samples with MARTi
Source: Genome Res. 2025 Nov;35(11):2488–500. doi: 10.1101/gr.280550.125 (PMC12581910; doi:10.1101/gr.280550.125)

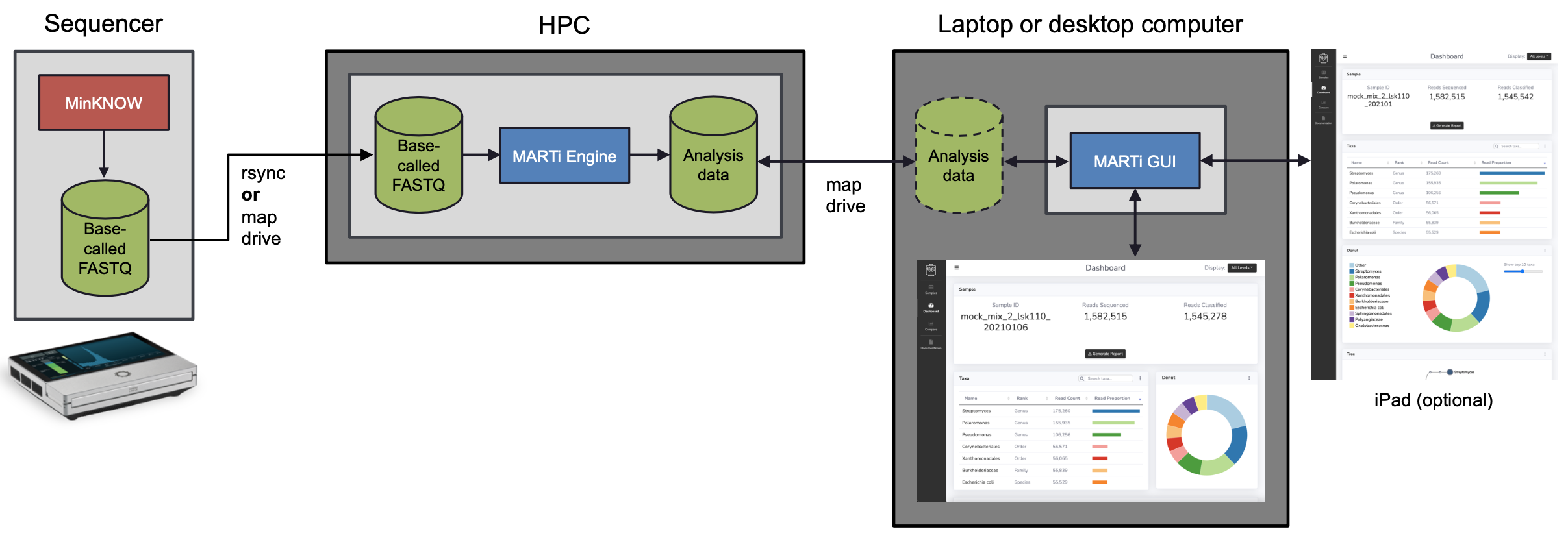

Supplement: Supplement 7 [file Supplemental_Code.zip › Supplemental_Code/MARTi-main/docs/source/images/ArchitectureHPC.png]

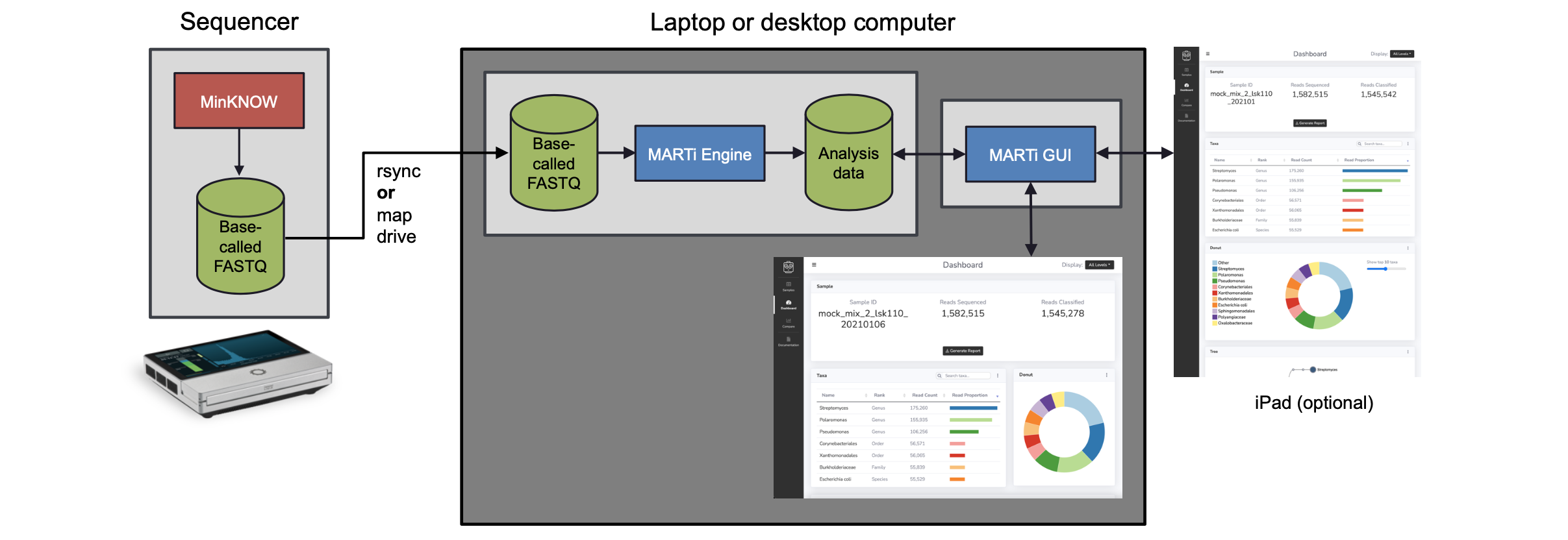

Supplement: Supplement 7 [file Supplemental_Code.zip › Supplemental_Code/MARTi-main/docs/source/images/ArchitecureLocal.png]

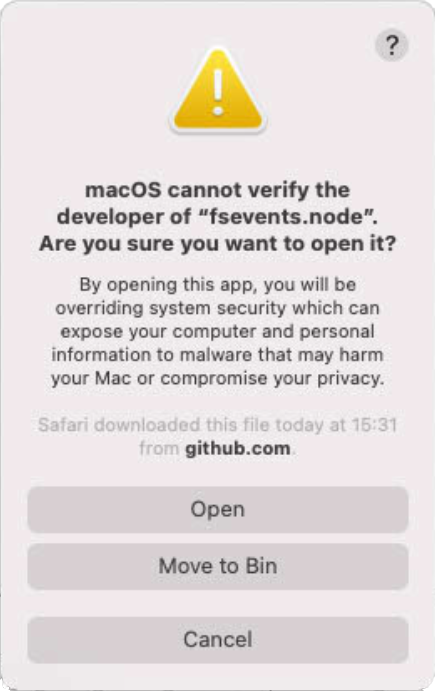

Supplement: Supplement 7 [file Supplemental_Code.zip › Supplemental_Code/MARTi-main/docs/source/images/fseventsdeveloper.png]

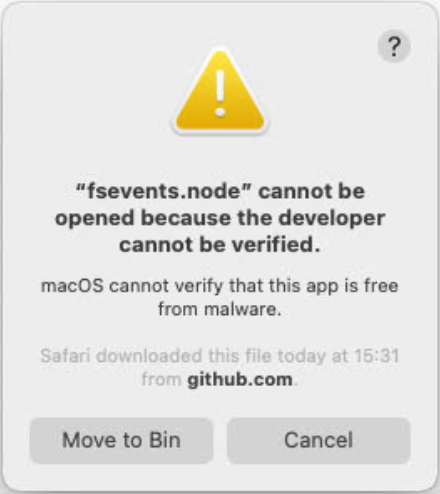

Supplement: Supplement 7 [file Supplemental_Code.zip › Supplemental_Code/MARTi-main/docs/source/images/fseventserror.png]

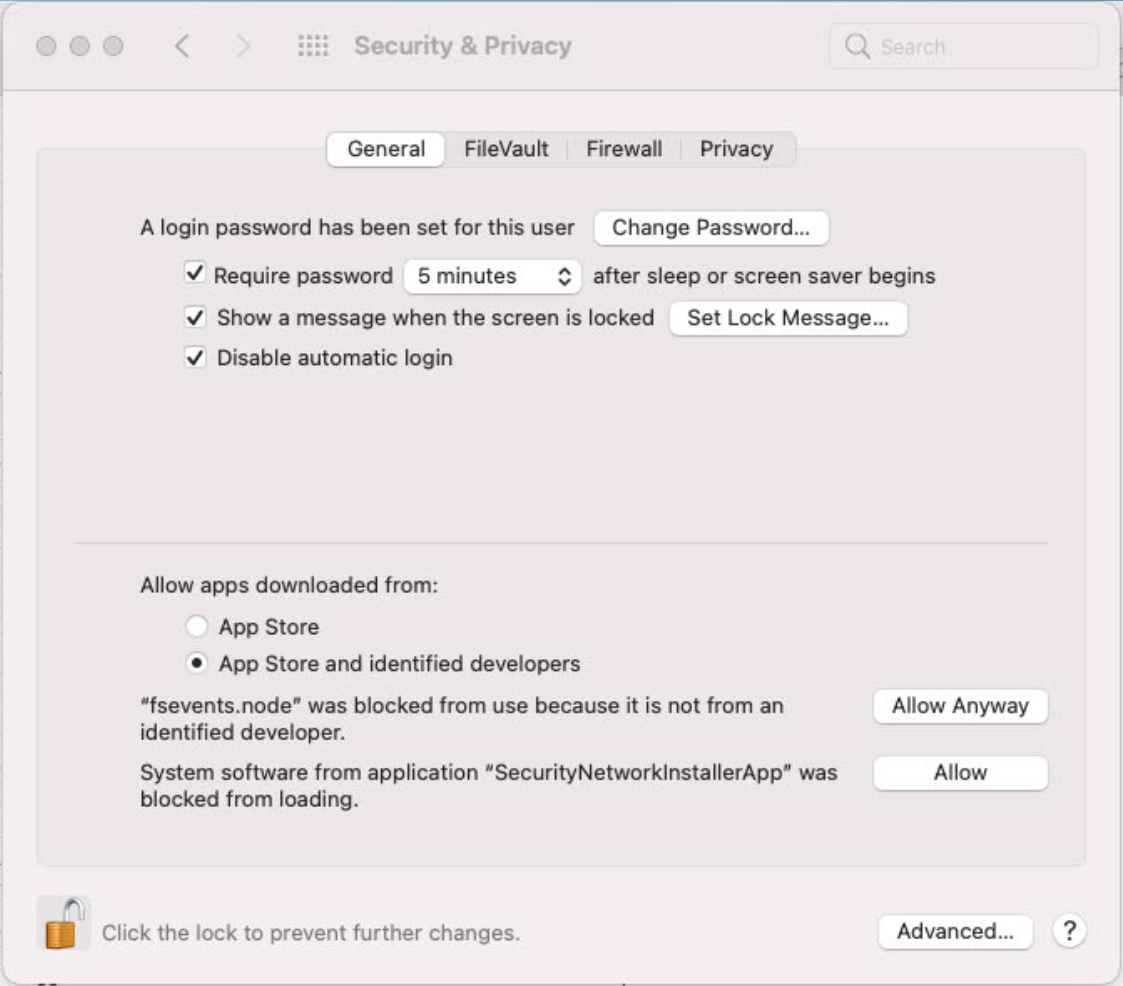

Supplement: Supplement 7 [file Supplemental_Code.zip › Supplemental_Code/MARTi-main/docs/source/images/fseventssecurity.png]

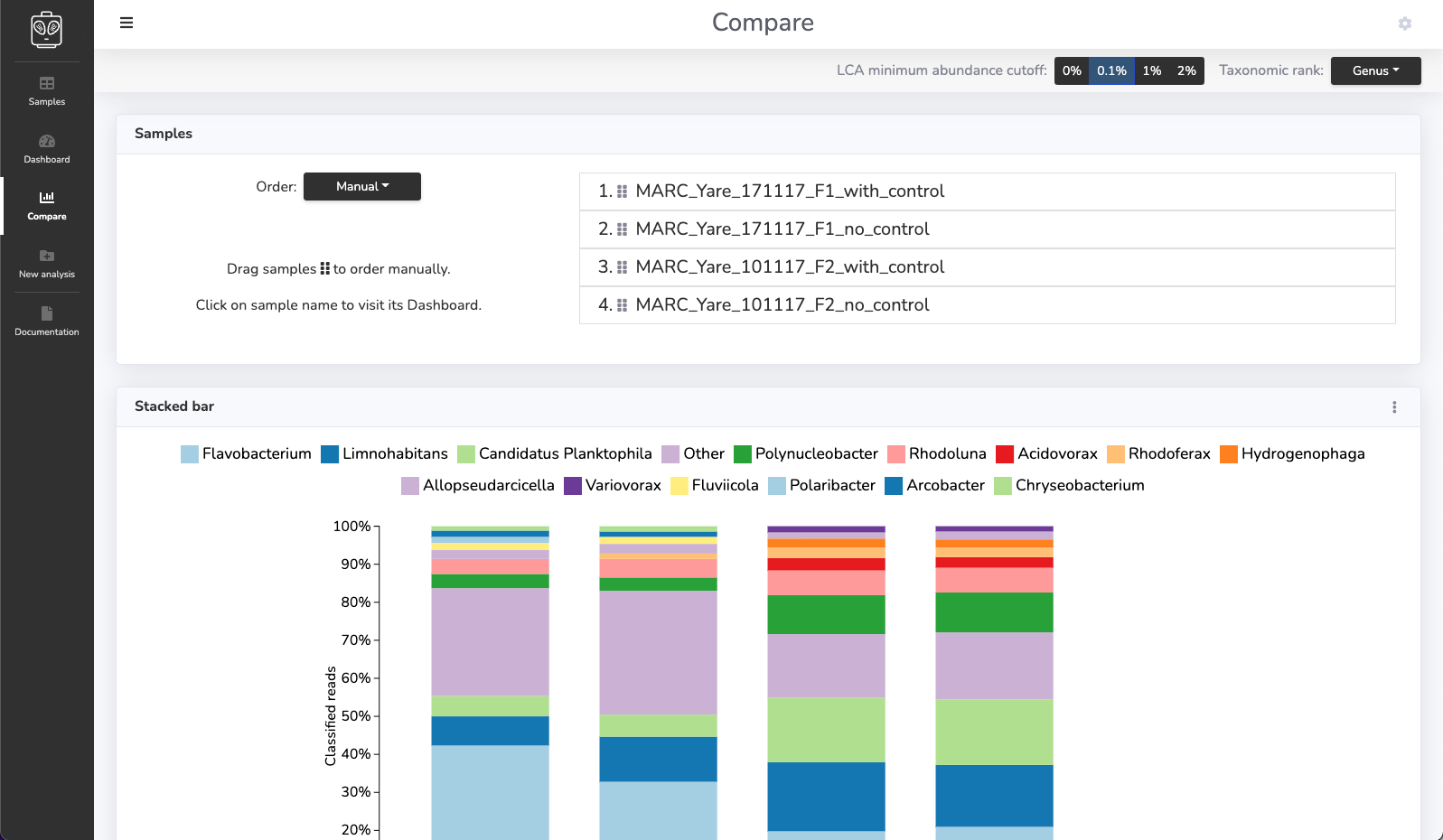

Supplement: Supplement 7 [file Supplemental_Code.zip › Supplemental_Code/MARTi-main/docs/source/images/GuiCompare.png]

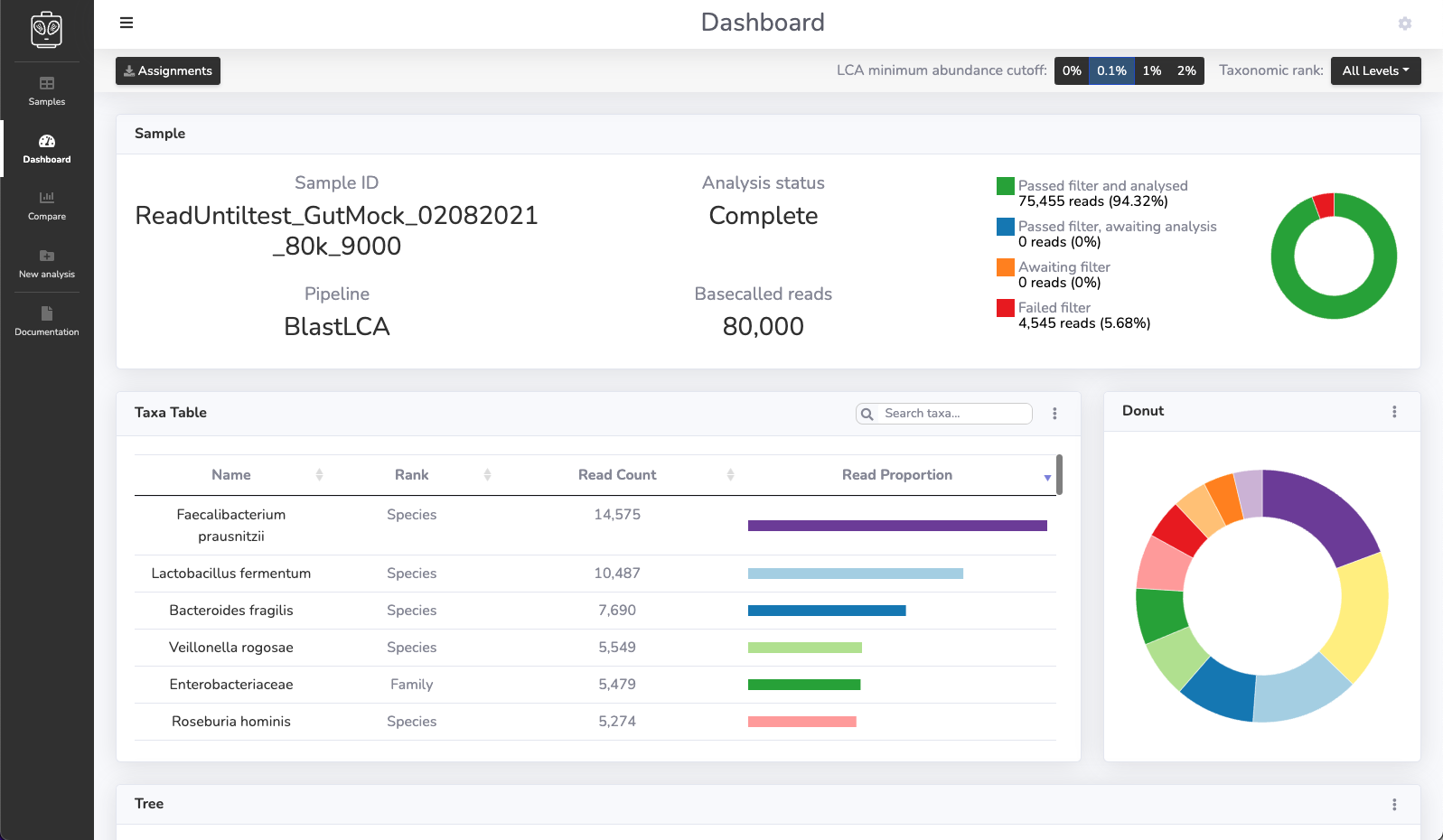

Supplement: Supplement 7 [file Supplemental_Code.zip › Supplemental_Code/MARTi-main/docs/source/images/GuiDashboard.png]

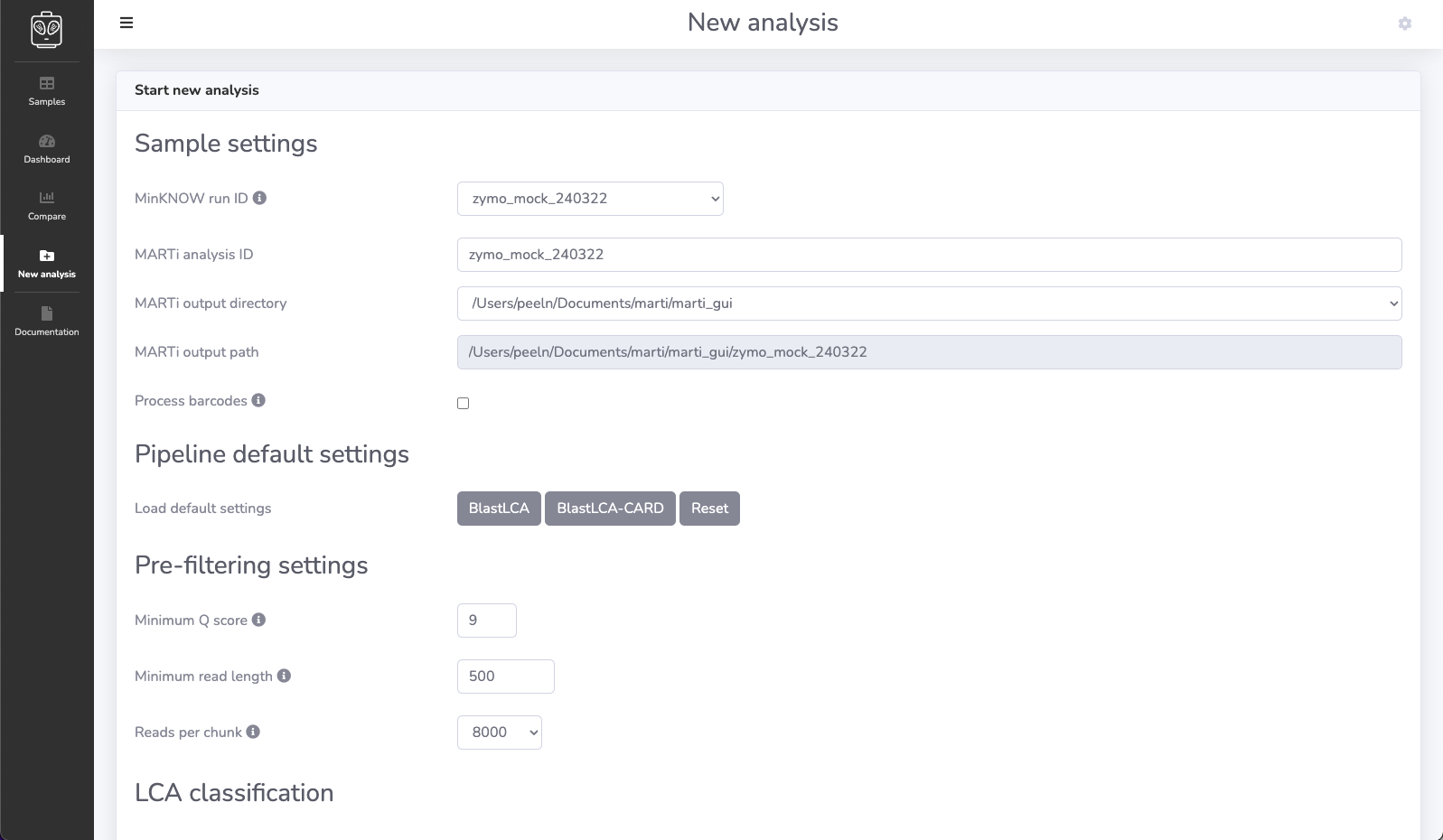

Supplement: Supplement 7 [file Supplemental_Code.zip › Supplemental_Code/MARTi-main/docs/source/images/GuiNew.png]

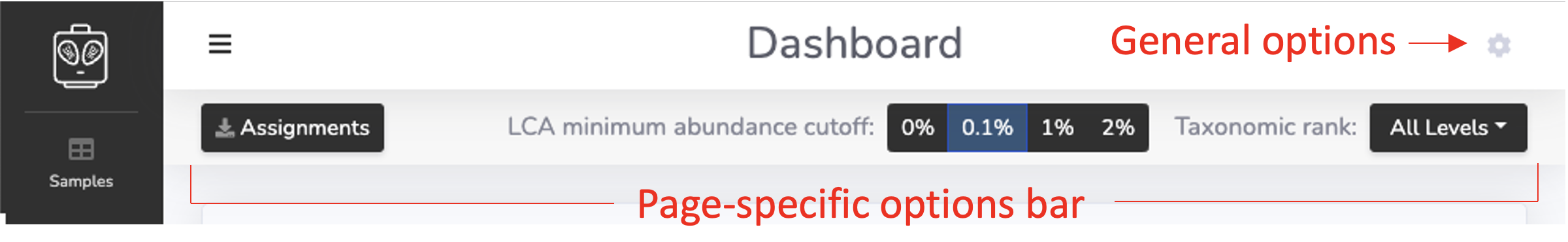

Supplement: Supplement 7 [file Supplemental_Code.zip › Supplemental_Code/MARTi-main/docs/source/images/GuiOptionsGeneral.png]

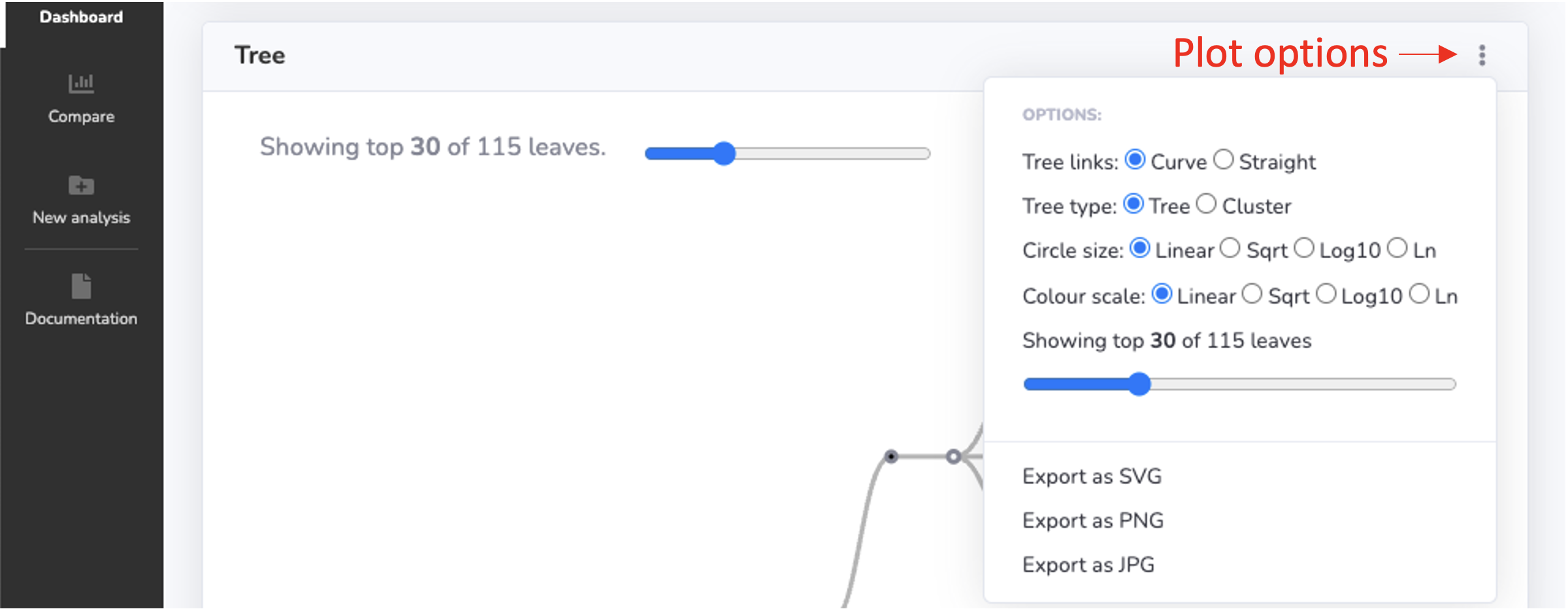

Supplement: Supplement 7 [file Supplemental_Code.zip › Supplemental_Code/MARTi-main/docs/source/images/GuiOptionsPlot.png]

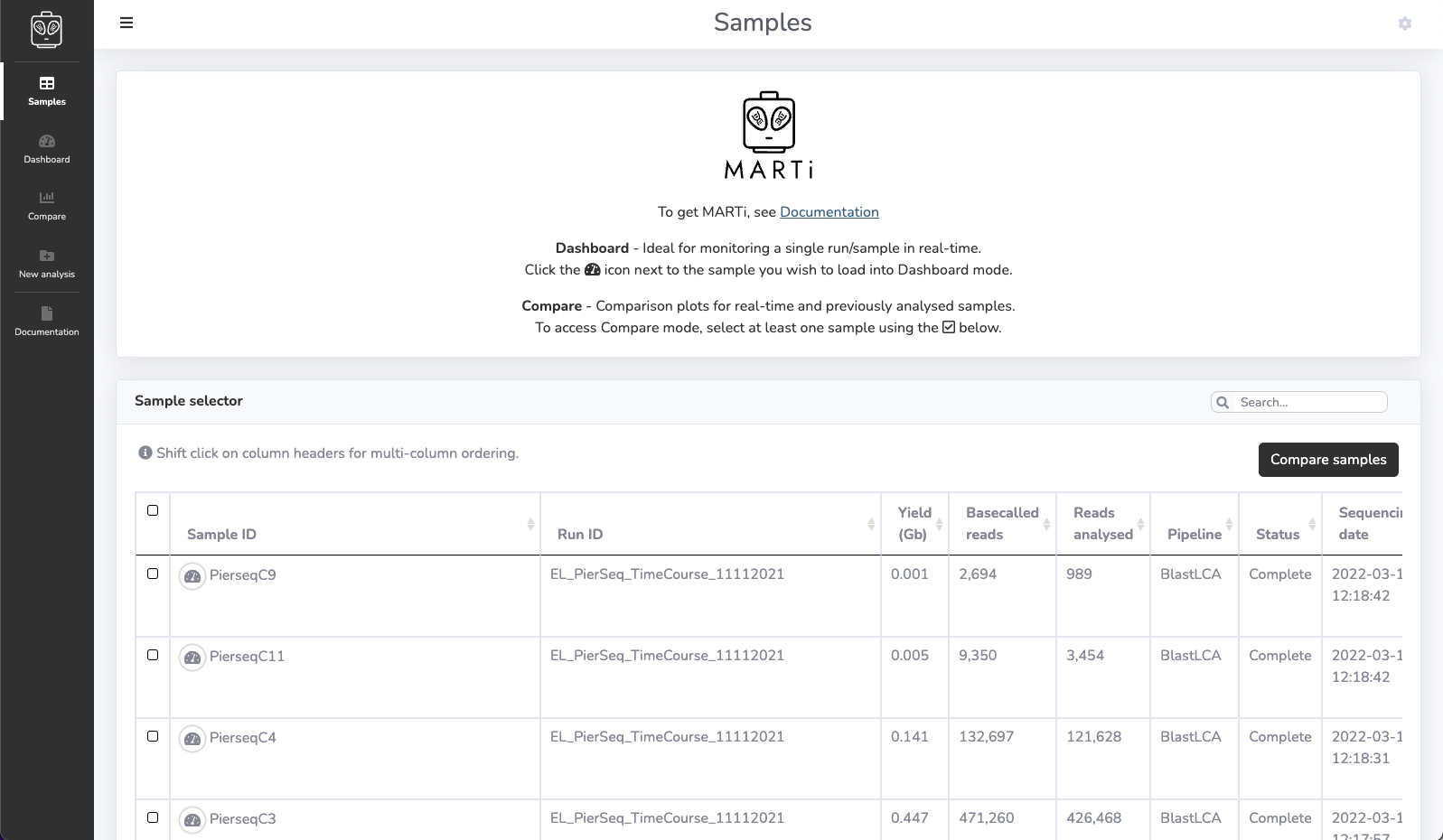

Supplement: Supplement 7 [file Supplemental_Code.zip › Supplemental_Code/MARTi-main/docs/source/images/GuiSamples.png]

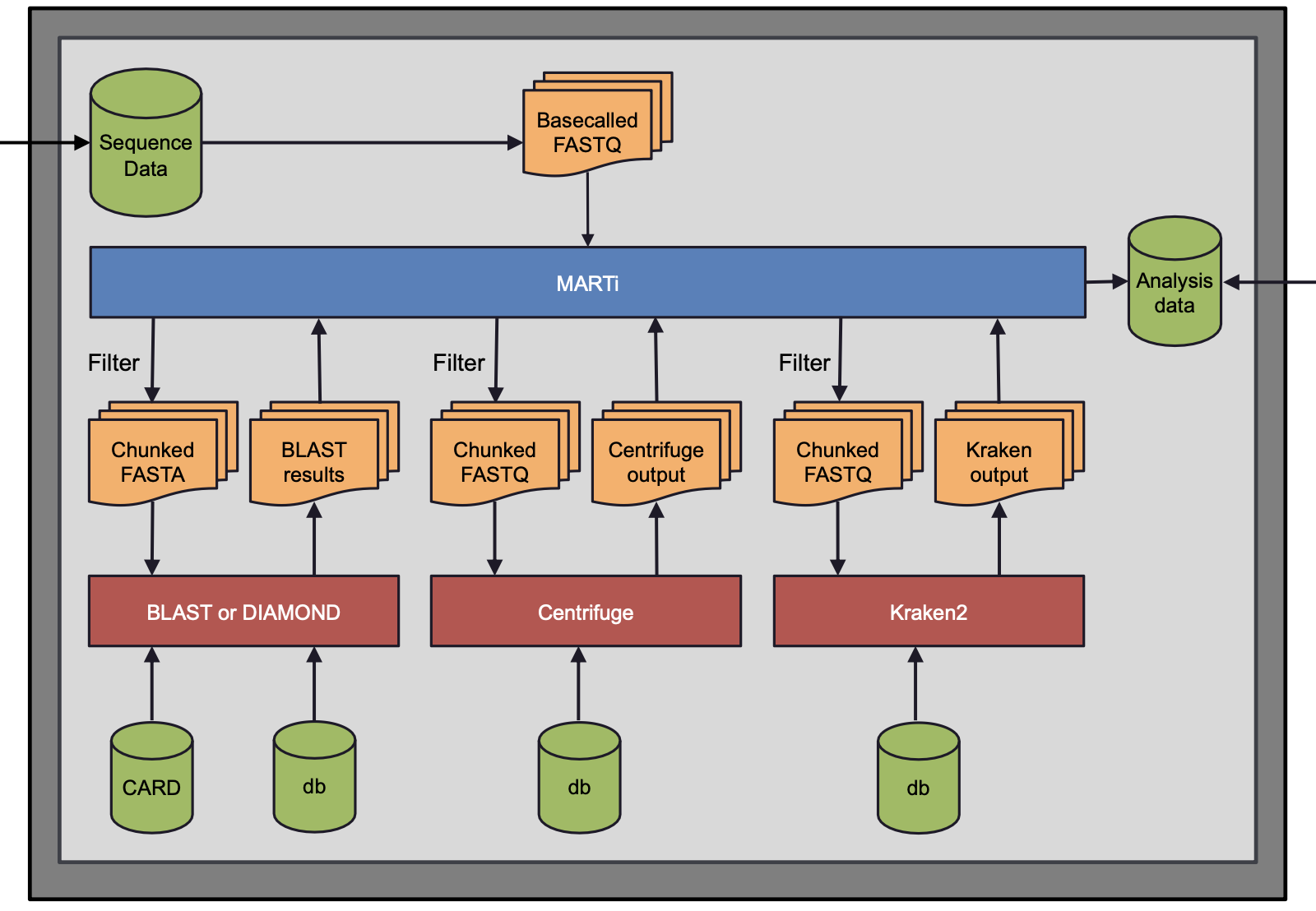

Supplement: Supplement 7 [file Supplemental_Code.zip › Supplemental_Code/MARTi-main/docs/source/images/InsideMARTiEngine.png]
