# Supplementary material for: Real-time analysis and visualization of nanopore metagenomic samples with MARTi
Source: Genome Res. 2025 Nov;35(11):2488–500. doi: 10.1101/gr.280550.125 (PMC12581910; doi:10.1101/gr.280550.125)
Supplement: Supplement 7 [file Supplemental_Code.zip › Supplemental_Code/MARTi-main/gui/UI/indexNode.html]

MARTi


---


- Samples
- Dashboard
- Compare
- New analysis


---

- Documentation


# MARTi

- !

  ###### Alerts

  No alerts


### To use MARTi, please enable JavaScript in your browser.


MARTi GUI v0.0.0
0


##### Options

×

Colour palette

Small (colour blind friendly) (9 colours)

Default (11 colours)

Medium (14 colours)

Large (21 colours)

Large (24 colours)

Close

##### You need to select a sample!

×

In order to access Dashboard mode, you must select a sample from the Sample selector table by clicking on the  icon next to the sample name.

Close

##### You need to select a sample!

×

In order to access Compare mode, you must select at least one sample from the Sample table by checkbox

Close

##### Page locked

×

Download and install MARTi to access the new analysis page.

Close

##### Portrait detected!

×

MARTi works best in landscape. Please rotate your device.

Close
