# Supplementary material for: Real-time analysis and visualization of nanopore metagenomic samples with MARTi
Source: Genome Res. 2025 Nov;35(11):2488–500. doi: 10.1101/gr.280550.125 (PMC12581910; doi:10.1101/gr.280550.125)
Supplement: Supplement 7 [file Supplemental_Code.zip › Supplemental_Code/MARTi-main/gui/UI/public/compare.html]

Assignments
LCA cutoff:
LCA minimum abundance cutoff:


0%
0.1%
1%
2%

Rank:
Taxonomic rank:


All Levels

All Levels
Domain
Phylum
Class
Order
Family
Genus
Species

Abundance:


Read count
bp yield

###### Samples

Order:


ID asc

ID asc
ID desc
Run asc
Run desc
Yield asc
Yield desc
Reads analysed asc
Reads analysed desc
Manual

Drag samples  to order manually.

Click on sample name to visit its Dashboard.

###### Stacked bar

Options:

Show top N taxa per sample

Y-axis:
 Count
 Percent

Unclassified node:
 Show
 Hide

Higher taxa node:
 Show
 Hide

Export as SVG
Export as PNG
Export as JPG

###### Donut

Options:


Show top N taxa per sample

Donut radius:

Count label:
 On
 Off

Sample name:
 Show
 Hide

Unclassified node:
 Show
 Hide

Higher taxa node:
 Show
 Hide


Export as SVG
Export as PNG
Export as JPG

###### Tree

Options:

Value:
 Percent
 Count

Value scale:
 Linear
 Sqrt
 Log10
 Ln

Tree type:
 Tree
 Cluster

Node width: N px

Node height: N px

Horizontal node position: N px

Show top N leaves per sample

Horizontal separation: N px

Vertical separation: N px

Sibling node separation: N

Cousin node separation: N

Export as SVG
Export as PNG
Export as JPG

### Generating tree...

###### Taxa heatmap

Options:

Show top N taxa per sample

Unclassified node:
 Show
 Hide

Higher taxa node:
 Show
 Hide

Colour palette:

Blue

Viridis

Magma

Grey zero:
 Off
 On

Value:
 Percent
 Count

Max cell width: N px

Export as SVG
Export as PNG
Export as JPG

###### Taxa accumulation

Options:

Curve:
 Linear
 Monotone

X-axis max: N

Export CSV

Export as SVG
Export as PNG
Export as JPG

When LCA minimum abundance cutoff is not 0%, curve may go down as well as up

### Data Not Available

#### Please run accumulation analysis to see this plot

###### AMR heatmap

Options:

Plot hits by

Gene
Drug class
Resistance mechanism

Show top N per sample

Colour palette:

Blue

Viridis

Magma

Grey zero:
 Off
 On

Value:
 Percent
 Count

Max cell width: N px

Export as CSV

Export as SVG
Export as PNG
Export as JPG
