# Supplementary material for: Real-time analysis and visualization of nanopore metagenomic samples with MARTi
Source: Genome Res. 2025 Nov;35(11):2488–500. doi: 10.1101/gr.280550.125 (PMC12581910; doi:10.1101/gr.280550.125)
Supplement: Supplement 7 [file Supplemental_Code.zip › Supplemental_Code/MARTi-main/gui/UI/public/dashboard.html]

Assignments
LCA cutoff:
LCA minimum abundance cutoff:


0%
0.1%
1%
2%

Rank:
Taxonomic rank:


All Levels

All Levels
Domain
Phylum
Class
Order
Family
Genus
Species

Abundance:


Read count
bp yield

Sample ID

Barcode

-

Pipeline

-

Engine version

-

###### Reads

---

Basecalled reads

##### -

Database

##### -

Classified reads

##### -

Unclassified reads

##### -

Basecalled yield

##### -

Classified yield

##### -

### These reads are being analysed...

Plots will appear after the first chunk of reads has been analysed.

###### Taxa Table

Export to CSV

###### Donut


Options:

Show top N taxa

Unclassified node:
 Show
 Hide

Higher taxa node:
 Show
 Hide

Export as SVG
Export as PNG
Export as JPG

Show top N taxa

###### Tree

Options:

Tree links:
 Curve
 Straight

Tree type:
 Tree
 Cluster

Circle size:
 Linear
 Sqrt
 Log10
 Ln

Colour scale:
 Linear
 Sqrt
 Log10
 Ln

Showing top N of N leaves

Export as SVG
Export as PNG
Export as JPG

Showing top N of N leaves

###### Treemap

Options:

Shape:
 Rectangle
 Square

Background colour:
 Black
 White

Unclassified node:
 Show
 Hide

Root node:
 Show
 Hide

Higher taxa node:
 Show
 Hide

Min font size: N px

Colour by:

Domain
Phylum
Class
Order
Family
Genus

Group label:
 Show
 Hide

Show only:

All
Bacteria
Eukaryota
Archaea
Viruses
Prokaryota

Export as SVG
Export as PNG
Export as JPG

Showing top N of N nodes

###### Sankey

Options:

Root node:
 Show
 Hide

Unclassified node:
 Show
 Hide

Show labels in columns:

R
 D
 K
 P
 C
 O
 F
 G
 S

Show columns:

D
 K
 P
 C 
 O 
 F
 G
 S

Export as SVG
Export as PNG
Export as JPG

Showing top 25
of 0 leaves

###### Taxa accumulation - All levels

Options:

Curve:
 Linear
 Monotone

X-axis max: N

Export CSV

Export as SVG
Export as PNG
Export as JPG

When LCA minimum abundance cutoff is not 0%, curve may go down as well as up

### Data Not Available

#### Please run accumulation analysis to see this plot


###### AMR Table

Options:


Export to CSV
Copy to clipboard

Chunk
1/1

1 Jan 2020

Toggle column:

Putative host taxa

Putative plasmid hits

Description

Resistance mechanism

Gene family

Drug class

###### Putative Host Taxa Analysis

Options:


Export as SVG
Export as PNG
Export as JPG

Chunk

1/1

1 Jan 2020

Plot taxa by

Show top N

AMR Gene

Drug Class

Resistance mechanism

###### AMR hits

Options:


Export as SVG
Export as PNG
Export as JPG

Chunk
1/1

1 Jan 2020

Plot hits by

Show top N

Show hits to

#### Click to add chart

##### Accumulation

Taxa accumulation plot.

##### AMR Table

Antimicrobial resistance analysis table.

##### Putative Host Analysis

Donut plot of taxa identified via AMR walkout analysis.

##### AMR Hits

Donut plot of AMR hits.

##### AMR gene information

×

Name:
:   name

Accession:
:   accession

Short name:
:   short name

---

Description:
:   description

---

Gene family:
:   n/a

Drug class:
:   n/a

Resistance mechanism:
:   n/a

URL:
:   url

Close
