# Supplementary material for: Real-time analysis and visualization of nanopore metagenomic samples with MARTi
Source: Genome Res. 2025 Nov;35(11):2488–500. doi: 10.1101/gr.280550.125 (PMC12581910; doi:10.1101/gr.280550.125)
Supplement: Supplement 7 [file Supplemental_Code.zip › Supplemental_Code/MARTi-main/gui/UI/public/footer.html]

Copyright © MARTi 2020
