# Supplementary material for: Real-time analysis and visualization of nanopore metagenomic samples with MARTi
Source: Genome Res. 2025 Nov;35(11):2488–500. doi: 10.1101/gr.280550.125 (PMC12581910; doi:10.1101/gr.280550.125)
Supplement: Supplement 7 [file Supplemental_Code.zip › Supplemental_Code/MARTi-main/gui/UI/public/new.html]

###### Sample settings

Input data directory

MARTi analysis ID

MARTi output directory

MARTi output path

Process barcodes

Select barcodes

###### Pre-filtering settings

Minimum Q score

Minimum read length

Reads per chunk

1000
2000
3000
4000
8000
16000
Other

###### LCA classification (BLAST only)

Max hits

20

Score percent

90 %

Minimum identity

70 %

Minimum query coverage

0 %

Minimum combined score

0

###### Scheduling

Inactivity timeout

Stop processing after

Max jobs

###### Analysis processes

Add processes

Start analysis
 Download config file
