# Supplementary material for: Real-time analysis and visualization of nanopore metagenomic samples with MARTi
Source: Genome Res. 2025 Nov;35(11):2488–500. doi: 10.1101/gr.280550.125 (PMC12581910; doi:10.1101/gr.280550.125)
Supplement: Supplement 7 [file Supplemental_Code.zip › Supplemental_Code/MARTi-main/gui/UI/public/samples.html]

For installation and usage instructions, see Documentation

Dashboard mode

Ideal for monitoring a single run/sample in real-time.

Click the  icon next to the sample you wish to load into Dashboard mode.

Compare mode

Comparison plots for real-time and previously analysed samples.

To access Compare mode, select at least one sample using the  below.

###### Export data

LCA minimum abundance cutoff:

0%
 0.1%
 1%
 2%

Taxonomic rank:

Columns:

Name

NCBI ID

Rank

Read count

Summed read count

Mean identity

Mean max identity

Field separator:

Comma
 Tab
 Colon
 Semicolon

###### Sample Map

Unstable internet connection detected!

###### Sample selector

Shift click on column headers for multi-column ordering.
Compare samples

|  | Sample ID | Run ID | Yield (Gb) | Basecalled reads | Reads analysed | Pipeline | Status | Sequencing date | Analysis date | More info |
| --- | --- | --- | --- | --- | --- | --- | --- | --- | --- | --- |
|  |  |  |  |  |  |  |  |  |  |  |
| --- | --- | --- | --- | --- | --- | --- | --- | --- | --- | --- |
|  |  |  |  |  |  |  |  |  |  |  |

Compare samples

##### Sample information

×

##### Sample

Name:


Run:
:   the\_run\_id

Sequencing date:
:   10/06/2022

Analysis date:
:   11/06/2022

##### Summary

Yield (Gb):
:   17.5

Basecalled reads:
:   127,893

Reads passed filter:
:   127,893

Reads analysed:
:   127,893

Reads classified:
:   127,893

Reads unclassified:
:   127,893

Reads with poor alignments:
:   127,893

##### Analysis

MARTi Engine version:
:   v0.9.10

Status:
:   Complete

Pipeline:
:   BlastLCA

Close

##### Please select at least one sample!

×

In order to export data, you must select at least one sample from the Sample selector table below using the checkboxes

Close
